# *Trichoderma and Bacillus* multifunctional allies for plant growth and health in saline soils: recent advances and future challenges

**DOI:** 10.3389/fmicb.2024.1423980

**Published:** 2024-08-08

**Authors:** Gustavo Santoyo, Ma. del Carmen Orozco-Mosqueda, Muhammad Siddique Afridi, Debasis Mitra, Eduardo Valencia-Cantero, Lourdes Macías-Rodríguez

**Affiliations:** ^1^Instituto de Investigaciones Químico Biológicas, Universidad Michoacana de San Nicolás de Hidalgo, Ciudad Universitaria, Morelia, Michoacán, Mexico; ^2^Departamento de Ingeniería Bioquímica y Ambiental, Tecnológico Nacional de México en Celaya, Celaya, Gto, Mexico; ^3^Department of Plant Pathology, Federal University of Lavras, Lavras, MG, Brazil; ^4^Department of Microbiology, Graphic Era (Deemed to be University), Dehradun, Uttarakhand, India

**Keywords:** abiotic stress, PGPR, fungi, *Bacillus*, endophytes

## Abstract

Saline soils pose significant challenges to global agricultural productivity, hindering crop growth and efficiency. Despite various mitigation strategies, the issue persists, underscoring the need for innovative and sustainable solutions. One promising approach involves leveraging microorganisms and their plant interactions to reclaim saline soils and bolster crop yields. This review highlights pioneering and recent advancements in utilizing multi-traits *Trichoderma* and *Bacillus* species as potent promoters of plant growth and health. It examines the multifaceted impacts of saline stress on plants and microbes, elucidating their physiological and molecular responses. Additionally, it delves into the role of ACC deaminase in mitigating plant ethylene levels by *Trichoderma* and *Bacillus* species. Although there are several studies on *Trichoderma-Bacillus*, much remains to be understood about their synergistic relationships and their potential as auxiliaries in the phytoremediation of saline soils, which is why this work addresses these challenges.

## 1 Introduction

Soil salinization is a growing global problem that affects plant growth, soil quality, and agricultural productivity. Salinity stress in agricultural lands predominantly arises from the accumulation of soluble salts. The main causes of salt stress include irrigation practices, poor drainage, and natural processes such as the weathering of rocks (Mohanavelu et al., 2021). Salinity stress adversely affects photosynthesis, respiration, and protein synthesis in plant cell (Afridi et al., 2019). Excessive salt concentrations impede water uptake by plant roots, leading to osmotic stress, disruption of the cellular water balance, and impaired nutrient absorption. These detrimental effects are further exacerbated by the induction of oxidative stress, disruption of cellular functions, and compromise of the plant's physiological processes, ultimately hindering their growth and yield (Zhang et al., 2016).

Salt is naturally present in the environment. However, poor management of agricultural fields might increase the salt content, such as incorrect irrigation management, inadequate application of manure and other animal waste, lack of soil drainage, and poor management of fertilizers, among others. The impact of salt stress on plants is multifaceted and involves disruptions in nutrient uptake, inhibition of photosynthesis, induction of oxidative stress, triggering the generation of reactive oxygen species, damaging cellular components, and interfering with essential metabolic processes (Horie et al., 2012; Ilangumaran and Smith, 2017).

Innovative technologies, including precision irrigation and the development of salt-tolerant crop varieties through molecular breeding, show promise for addressing the complexities of saline soil agriculture. Nevertheless, the multifaceted nature of saline soil challenges underscores the importance of holistic approaches that integrate soil management practices, crop selection, and emerging technologies (Afridi et al., 2019). Plant-microbe interactions have a significant impact on the ability of plants to tolerate salt stress. Microbial communities found in association with plant roots play a vital role in promoting plant growth and maintaining plant health. These communities are often referred to as the “second genome” of plants (Berendsen et al., 2012; Moreira et al., 2020).

Microbial-assisted strategies have emerged as promising tools for bioremediation and promotion of agricultural production in saline soils (Mitra et al., 2021). Beneficial microbes, including bacteria and fungi, play pivotal roles in enhancing plant resilience to stressful conditions. These microbes establish symbiotic relationships with plants, foster nutrient uptake, modulate hormone levels, produce osmoprotectants, and activate stress response mechanisms. Phytoremediation is an environmentally friendly approach to remediating saline soils (Jesus et al., 2015). It involves growing plants that can accumulate or tolerate salt and has a history of reducing salinity concerns. The two primary mechanisms involved are based on either the ability of the root to prevent salt infiltration or the regulation of salt concentration and distribution. The plant species employed for phytoremediation primarily consist of halophytes, hyperaccumulators, salt-tolerant plants, and transgenic plants (Zhang S. et al., 2023). Phytoremediation is a green and environmentally friendly technique that harnesses the inherent capabilities of plants and their associated microbes to remediate saline soils (Mujeeb et al., 2023).

Plant growth-promoting rhizobacteria enhance plant growth and salinity resistance, while halotolerant bacteria boost growth under saline stress through direct or indirect mechanisms (Khoshru et al., 2020). Other beneficial fungi, such mycorrhizal fungi (AMF) enhance plant resistance to salinity stress by improving nutrient uptake, water absorption, and osmolytes accumulation. For example, osmolytes like proline help organisms tolerate salinity primarily by maintaining osmotic balance, stabilizing proteins, and supporting redox potential (Hanin et al., 2016). In addition, the proper cultivation of salinity-tolerant plants can improve calcium levels and decrease sodium levels in the soil to improve these types of agricultural soils. After improving the soil, it is possible to plant crops with low salt tolerance assisted by microorganisms. Plants, known as hyperaccumuwithlators, in collaboration with beneficial microbes, facilitate the accumulation of these toxic elements, making the agro-environment less harmful (Berni et al., 2019). This synergistic relationship between plants and microbes not only aids in the detoxification of soils but also enhances the overall health and productivity of crops (Kapadia et al., 2021).

The intricate mechanisms involved in microbial-assisted strategies provide a multifaceted approach for mitigating the impact of salinity stress. Microbes contribute to salinity stress mitigation by promoting an ion balance within plant cells. Additionally, microbial communities enhance soil structure and water-holding capacity, mitigating the adverse effects of salinity on plant water availability (Loganathachetti et al., 2022). The co-occurrence of these stressors in saline soils exacerbates their detrimental effects on plant growth and productivity. The intricate interplay between salinity and other environmental stresses further complicates mitigation strategies and demands innovative approaches for sustainable agriculture (Hasanuzzaman and Fujita, 2022).

In recent years, microbial-assisted strategies have emerged as promising solutions for both phytoremediation and the enhancement of agricultural production in saline environments (Kumar A. et al., 2020). Microorganisms play a pivotal role in facilitating plant adaptation to stressful conditions by promoting nutrient uptake, modulating hormonal balance, and inducing stress-responsive pathways. This symbiotic relationship between plants and microbes has garnered attention as a potential tool to alleviate the deleterious effects of salinity on crop plants. Nevertheless, challenges such as microbial survival, competition, and scalability under field conditions persist, emphasizing the need for continued research and development (Mahmood et al., 2016; Sodhi and Saxena, 2023). This study explored the basic survival mechanisms of soil- and plant-beneficial microbes, mainly *Trichoderma* and *Bacillus* species, as well as the integration of microbial and plant-assisted strategies that hold the potential to address these challenges and further improve the efficacy of increasing agro-productivity in salt-affected soils. Likewise, to have a comprehensive vision of the potential of these two microbial groups not only in promoting plant growth, this work explores their role as remediators of saline soils.

### 1.1 Impact of salt stress on plants

Salt stress is a major challenge in plants. Salt diminishes the water potential and, in this way, complicates water absorption by plants, produces osmotic stress, reduces nutrient uptake and carbon fixation, produces an imbalance in ionic contents, damages enzymatic activity, disrupts the energy production apparatus electron transport chains, generates reactive oxygen species (ROS), and subsequently nitrosative and peroxidative lipid stress (Atta et al., 2023). Each of these processes negatively affects the health and growth of plants, and together, according to stress intensity, can lead to plant death.

### 1.2 Osmotic stress

Water potential is a function of water purity; as higher purity increases the water potential, water flows from high water potential to low water potential compartments, and the difference in water potential is the force that drives water flow from soil to plant roots (Robbins and Dinneny, 2015). Salt ions (Na^+^ Cl^−^ and other salt ions) diminish the water potential and thus reduce or even avoid water flow from the soil to the plant cells, leading to a water deficit (Sheldon et al., 2017). When a plant is in a situation where a water deficit limits its growth and development, it is referred to as osmotic stress. Osmotic stress affects plants at cellular, morphological, and anatomical levels. Cell turgor depends on osmotic pressure (Ali et al., 2023), and the elongation of cells and organs is strongly affected by the loss of cell turgor (Touati et al., 2015). Loss of cell turgor also leads to leaf wilting, a condition that reveals a critical water status in plants; a prolonged deficit of water can lead to leaf death (Babalik and Baydar, 2024). To prevent desiccation, plants reduce water transpiration by closing their stomatal pores (Hasanuzzaman et al., 2021), this response also reduces CO_2_ supply and photosynthesis (Talaat, 2021).

### 1.3 Ion toxicity and nutrient imbalance

In addition to osmotic stress, salt is toxic to plants. Na^+^ and Cl^−^ are not essential nutrients for plants. At high soil salt concentrations, Na^+^ penetrates the plant root through the root cortex and crosses the apoplastic barriers to the central cylinder of the vascular system (Krishnamurthy et al., 2011). Solutes traverse the plasma membrane; the influx of Na^+^ occurs mainly through non-selective cation channels (NSCCs), but members of transport families such as HKTs and AKT are also involved (Akhter et al., 2022). The influx of Na^+^ into the cytoplasm modifies the charge balance, promoting membrane depolarization and subsequent K+ efflux (Zhang X. et al., 2023).

More than 60 enzymes involved in plant primary metabolism, including catabolic and sugar metabolism, anabolic and protein synthesis, and photosynthesis, depend on K+ concentrations for their activation owing to indirect regulatory mechanisms or direct effects derived from the role of K+ as an enzyme cofactor. Consequently, a decrease in K+ concentration impulse due to Na^+^ influx results in toxic malfunctioning of plant metabolism (Slabu et al., 2009).

Virtually all the nutrients (Fe, K, Mn, Mg, P, Zn, Bo, and Cu) increase shoots and roots under salt stress conditions (Hussain et al., 2018); this is explicable by an increase in levels of Na+ and Cl^−^ ions in plant tissues, primarily affecting the content of other nutrients by ion competence, and because the scarcity of nutrients affects normal plant root development, lowers root hair density, and subsequently affects nutrient absorption (Atta et al., 2023).

### 1.4 Effect on photosynthesis

Salt stress exerts a robust inhibitory effect on photosynthesis. The osmotic stress produced by salt induces stomatal closure via the abscisic acid regulatory pathway to reduce water loss (Roychoudhury et al., 2013). In this way, salt stress strongly deprives stomatal conductance, which estimates not only the flux of water from leaf mesophyll tissue through the stomatal aperture to the atmosphere but also the flux of CO_2_ from the atmosphere to mesophyll tissue (Damour et al., 2010). Stomatal conductance is one of the most critical parameters for CO_2_ fixation via photosynthesis because it limits the CO_2_ supply for the Calvin-Benson cycle (Sakoda et al., 2021).

Salt stress also inhibits photosynthesis via non-stomatal conductance. These include alteration of photosynthetic pigment biosynthesis, inhibition of the Calvin-Benson cycle enzymes, and disruption of the integrity and efficiency of the photosynthetic apparatus and thylakoid membranes (Sharma et al., 2020).

### 1.5 Oxidative stress

As has been established, osmotic stress causes stomatal apertures to close to prevent desiccation and consequently reduces CO_2_ flux, thereby reducing CO_2_ levels (Ueda et al., 2013). Under these conditions, and if plants are exposed to sunlight, electrons from photosystems PSI and PSII are derived to molecular oxygen, resulting in the generation of ROS, at the same time, a decrease in K+ intracellular concentration depresses the antioxidant system, contributing to ROS increase (Ahanger and Agarwal, 2017). Increased ROS levels cause oxidative stress and induce DNA damage and peroxidation of membrane lipids, with concomitant damage to the photosynthetic apparatus (Hasanuzzaman and Fujita, 2022).

## 2 Impact of salt stress on soil microbes

Although some plants have evolved to adapt to saline environments, most are considered salt-sensitive. Halophytes are plants capable of completing their life cycle with at least 70 mM salt (Cheeseman, 2015) and are characterized as possessing physiological adaptations to counteract the effects of the presence of salt ions. Halophytes are not a monophyletic group; halophily has evolved in 37 orders of plants no fewer than 59 times (Ashraf and Munns, 2022), showing that the development of strategies to adapt to high salt content is based on the general physiological capabilities of plants. Strategies to adapt to high soil salinity include biosynthesis and accumulation of compatible solutes, upregulation of the antioxidant system, and toxic ion compartmentalization (Al-Turki et al., 2023).

### 2.1 Biosynthesis and accumulation of compatible solutes

Compatible solutes are small, osmotically active molecules that can accumulate at high concentrations with minor or non-adverse effects on cellular metabolism because of their hydrophilicity, and at least some of them can replace water at the surface of membranes and proteins (Zulfiqar et al., 2019). Compatible solutes drop the water potential inside the cell, allowing water influx, preventing water efflux, and protecting the cell from osmotic shock (Ozturk et al., 2021). The chemical nature of compatible solutes is diverse but they can be categorized as amino acids, betaines, non-reducing sugars and polyols, and polyamines (Kumar and Verma, 2018).

Under salt stress, spinach (*Spinacia oleracea*) accumulates glycine, serine, proline, and glycine betaine as free amino acids in its leaves. Glycine betaine comprises between 15% and 55 percent of the total nitrogen osmolytes, depending on the intensity of salt stress (Di Martino et al., 2003). It has been shown that rice, most saline-tolerant varieties, accumulates higher concentrations of proline with a significantly lower level of lipid peroxidation (Ha-tran et al., 2021), and similar results have also been observed in wheat (Hinai et al., 2022). Other frequently accumulated compatible solutes in plants under salt stress are sorbitol, mannitol, putrescine, spermidine, and spermine (Hassan et al., 2016; Chang et al., 2019; Islam et al., 2020).

### 2.2 Toxic Ion homeostasis and compartmentalization

Once Na^+^ and Cl^−^ enter the plant cell, the plant employs two different mechanisms to limit the deleterious effects of the ions. First, Na^+^ bombs back outside the cell to the apoplast or leaf surface, and second, the plant compartmentalizes the toxic ion in the vascular space or even in the old leaves (Keisham et al., 2018).

The plasma membrane Na^+^/H^+^ antiporter salt-overly-sensitive 1 (SOS1) is a major player in salt tolerance, and SOS1 excludes cytosolic Na^+^ from the apoplast (Zhang X. et al., 2023). Additionally, SOS1 is involved in Na^+^ loading into the xylem, and rice *sos1* mutants are very salt sensitive (Akhter et al., 2022; Zhang X. et al., 2023). Yang et al. (2021) showed that transgenic lines of *Arabidopsis* plants, callus of *Malus domestica* (apple), and *M. domestica* plants expressing or overexpressing the Cation/Ca2C Exchanger 1 from *M. domestica* (MdCCX1) significantly increased tolerance to salt treatment, reducing the Na^+^ content and promoting ROS scavenging, indicating that MdCCX1 excludes Na^+^ from the plant.

The ability of plant cells to compartmentalize Na^+^ and Cl^−^ in vacuoles is important for maintaining low cytosolic toxic ion levels (Saghafi et al., 2020). NHXs Na^+^/H^+^ antiporters are mainly localized in the tonoplast membrane and play a central role in compartmentalizing Na+ in the vacuolar space, allowing the maintenance of higher concentrations of K+ (Heidari and Golpayegani, 2012; Keisham et al., 2018). The expression of *PutNHX1* and *SeNHX1* from the halophytes *Puccinellia tenuiflora* and *Salicornia europaea* confers the ability of Arabidopsis root cells to sequester large quantities of Na^+^ in the vacuolar space than controls and a concomitant higher cytosolic K+ accumulation (Liu et al., 2021), in the same sense overexpression of *SeNHX1* improves salt tolerance in tobacco (Chen et al., 2015).

### 2.3 Antioxidant system

Plants respond to oxidative stress produced by salt via the disruption of electron transport chains by upregulating their antioxidant systems (Hasanuzzaman et al., 2021). In addition to compatible solutes, plants produce a wide range of antioxidant compounds, such as polyphenols, flavonoids, carotenoids, and glycoalkaloids (Kiani et al., 2021).

Other compounds that accumulate under salt stress are polyamines, which act as compatible solutes that act as antioxidant agents and are involved in the regulation of enzymatic antioxidant plant systems (Saha et al., 2015; Gowtham et al., 2022). ROS produced under salt stress are abated by antioxidative enzymes, including superoxide dismutase, glutathione reductase, dihydroascorbate reductase, ascorbate peroxidase, catalase, and guaiacol peroxidase (Zhang et al., 2016). Exogenous application of the polyamines putrescine, spermidine, and spermine to mung bean (*Vigna radiata*) grown under salt treatment enhanced ascorbate and glutathione content and increased the activities of the antioxidant enzymes dehydroascorbate reductase, glutathione reductase catalase, and glutathione peroxidase. Similarly, the exogenous application of phenanthroline, a polyamine synthesis inhibitor, significantly inhibits the activity of superoxide dismutase, catalase, ascorbate peroxidase, and monodehydroascorbate reductase in tomato plants under salt stress (Zhong et al., 2020).

## 3 Impact of salt stress on soil microbes

Soil microorganisms have evolved mechanisms to tolerate different salt concentrations in the environment, such as saline soils, some of which are used for agricultural production purposes. Microorganisms can be classified on the basis of their salt tolerance. For example, halotolerant organisms can survive in environments containing up to 25% sodium chloride, whereas halophiles not only tolerate salt but also require it to grow. Within this broad classification of halotolerant and halophilic organisms, there are further divisions that identify microorganisms as non-halophiles ( ≤ 1%), slight (1%−3%), moderate (3%−15%), or extreme halophiles (≥15%−25%) (Kanekar et al., 2012).

In recent studies (Etesami and Emami, 2023; Etesami et al., 2023; Petrosyan et al., 2023), several bacterial genera have been listed as good salt tolerant candidates with the ability to associate with plants to promote growth and production under adverse conditions. Some examples include *Bacillus* spp., *Halomonas* spp., *Pseudomonas, Streptomyces* spp. Although some soil bacterial genera, such as Rhizobium, do not tolerate low concentrations of salt (do not grow in media such as Luria bertani with 0.5%−3% NaCl), variations exist among strains or species of rhizobia with salt tolerance. Some strains, such as *Mesorhizobium alhagi* CCNWXJ12-2, are highly salt-tolerant and capable of nodulating the desert plant, Alhagi sparsifolia. Through transcriptomic analysis of strain CCNWXJ12-2, possible genes such as *proV, proW, proX*, and *nhaA*, which encodes for the glycine-betaine transport system, an osmoprotectant. This system is crucial for accumulating compatible solutes inside the cell, which helps balance osmotic pressure without interfering with cellular metabolism. *proV* encodes an ATPase protein that provides energy for the active transport of glycine-betaine. *proW* encodes a protein that is part of the glycine-betaine transporter, while *proX* encodes the protein that acts as the glycine-betaine transporter across the membrane. Finally, the *nhaA* gene encodes for a Na+/H+ antiporter. This protein helps pump sodium ions (Na+) out of the cell and pump protons (H+) into the cell. However, deletion mutant analysis identified the *yadA* gene as being relevant for salt tolerance. The *yadA* gene encodes for an adhesin A, which in pathogens like Yersinia has virulence functions, but also plays a role in autoaggregation and protection. Additionally, this adhesin helps bacteria adhere to host cells and tissues, facilitating rhizosphere colonization (Liu et al., 2014).

The authors concluded that genes with functions such as osmoprotectant uptake and ion transporters are two of the most relevant mechanisms in the rhizobia *M. alhagi* to cope with salt stress. Strains such as CCNWXJ12-2 may have a high value in the bioinoculant market to stimulate production processes in naturally occurring host plant species, in addition to significant potential to explore their nodulation capacity with other agriculturally important plant species grown in saline soils.

Salinity stress can affect the viability of microorganisms in various ways, primarily by affecting the osmotic balance. If cells can regulate this effect and maintain internal ionic osmoregulation, they can tolerate salt stress. Therefore, halotolerant microorganisms typically contain genetic systems in their genomes that code for intracellular ion regulation systems by pumping ions out of the cell using plasma membrane-localized Na+/H+ antiporters, and they also make use of K+/Na+ ion transporters. The importance of Na+/H+ antiporters has been demonstrated for decades in species such as *Bacillus* (Whatmore et al., 1990); however, research has focused more on the functions of these antiporters in model plants such as Arabidopsis. Recently, Baek et al. (2020) showed that the SOS1 gene, encoding a Na+/H+ antiporter, is relevant for tolerating salt stress induced by interaction with the bacterium *Bacillus oryzicola* YC7007 in *Arabidopsis thaliana*. Interestingly, other mutant plants in sos2-1 and sos3-1 inoculated with YC7007 continued to show salt tolerance (but not when mutating the sos1-1 copy), indicating no function in halotolerance. Additionally, strain YC7007 showed beneficial effects by inhibiting the accumulation of malondialdehyde and Na+ induced by salt in seedlings co-cultured with YC7007. Moreover, other crop species, such as radish and cabbage, benefited from their interaction with plant growth-promoting rhizobacteria (PGPR), suggesting a potential for stimulating salt stress tolerance through this approach.

The cell membrane and cell wall are the main barriers that delimit the cellular interior and protect the cell from the external environment, including salt stress. Their components must be sufficiently “selective” to allow the entry of nutrients, ions, and important metabolites for growth while simultaneously preventing the passage of elements, compounds, or toxic substances. Therefore, one of the mechanisms of halotolerance involves the presence of membrane components such as phospholipids, fatty acids, glucans, and polysaccharides, among others, in “appropriate” proportions. Microorganisms can modulate their membrane components when they are exposed to saline stress. This was recently demonstrated by Rojas-Solis et al. (2020) for beneficial bacteria of the *Bacillus* spp. genus Bacillus. Under saline conditions, these bacteria modify the production of lipids and membrane fatty acids, allowing them to maintain their antagonistic mechanisms (e.g., antifungal activity against Botrytis cinerea) and promote growth in tomato plants (*Lycopersicon esculentum* “Saladette”). This includes the production of siderophores and indole-3-acetic acid (IAA), protease activity, and biofilm formation.

In a recent study by the same authors (Rojas-Solis et al., 2023), through the analysis of mutants of *clsA* (UM270 Δ*clsA*) and *clsB* (UM270 Δ*clsB*) in the biocontrol and plant growth-stimulating rhizobacterium *Pseudomonas fluorescens* UM270, cardiolipin membrane phospholipids were identified as relevant for salt stress tolerance. It was also found to be crucial for promoting the growth of *Lycopersicon esculentum* and saladette plants. During the experiments, it was observed that the UM270 strain significantly increased the production of cardiolipin under saline conditions (200 mM NaCl), whereas phosphatidylcholine decreased its production, and other phospholipids such as phosphatidylglycerol and phosphatidylethanolamine remained unchanged. Interestingly, mutants with reduced cardiolipin production failed to stimulate tomato growth under saline conditions. In contrast, the wild-type UM270 strain not only stimulated parameters such as shoot length, chlorophyll content, and total plant dry weight but also maintained active mechanisms such as the production of indole acetic acid, siderophores, and biofilm.

## 4 The role of ACC deaminase in salt-tolerant microbes

The mass production processes of agricultural products have increased soil salinization, such as irrigation with water high in salt content (Singh, 2021; Du et al., 2023). Therefore, when attempting to apply any biological inoculant, which may include fungi, bacteria, or both microorganisms, namely plant growth-promoting microbes or PGPM, they must exhibit survival mechanisms to tolerate salt stress. Some mechanisms include the production of osmolytes, improving responses to reactive oxygen species (ROS) production, or modifying the components of cell membranes. All these mechanisms have been recently analyzed and discussed by Valencia-Marin et al. (2024), so reading their work is suggested. Here, we present a brief analysis of the role of microbial ACC deaminase in reducing ethylene levels in plants affected by saline stress, which is significant in PGPM, such as *Trichoderma* and *Bacillus*, for effectively promoting plant growth and health in multiple crops.

### 4.1 Ethylene

One of the mechanisms that plant-associated microorganisms use to help tolerate salt, as well as enhance host growth and development, is 1-aminocyclopropane-1-carboxylic acid (ACC) deaminase activity (Gamalero et al., 2023). Briefly, and as explained earlier, when plants are subjected to salt stress (among other stress types such as drought or flooding, exposure to heavy metals, etc.), ethylene synthesis increases in the tissues. Thus, ethylene synthesis begins with the production of S-adenosyl-methionine (SAM), a reaction catalyzed by the enzyme SAM synthase, derived from the combination of methionine and ATP. Subsequently, SAM is converted to ACC by the enzyme ACC synthase, and in turn, ACC is a direct derivative of ethylene (ET). This latter reaction is carried out by the enzyme ACC oxidase. As a result of this reaction, some volatile compounds are produced, such as CO_2_ and Hydrogen Cyanide or HCN (Orozco-Mosqueda et al., 2020). It is worth mentioning that some effects of excessive ethylene production under stress conditions can lead to chlorosis and/or foliar necrosis, rapid aging and wilting of flowers or leaves, or cause deformation in flowers, premature leaf or flower drop (abscission), and reduced growth of plants and fruits. Ethylene can induce seed germination, elongation of plant roots, formation of leaf and root primordia in stems and roots, and initiation of flowering. In fruits and vegetables, ethylene can induce ripening and accelerate product rotting (Zhang et al., 2020). Therefore, it is necessary to keep ethylene levels low in crops with the intention of promoting seed germination, the formation of leaf and root primordia in stems and roots, as well as the elongation of plant roots and initiation of flowering.

### 4.2 ACC deaminase model

**Figure 1:**
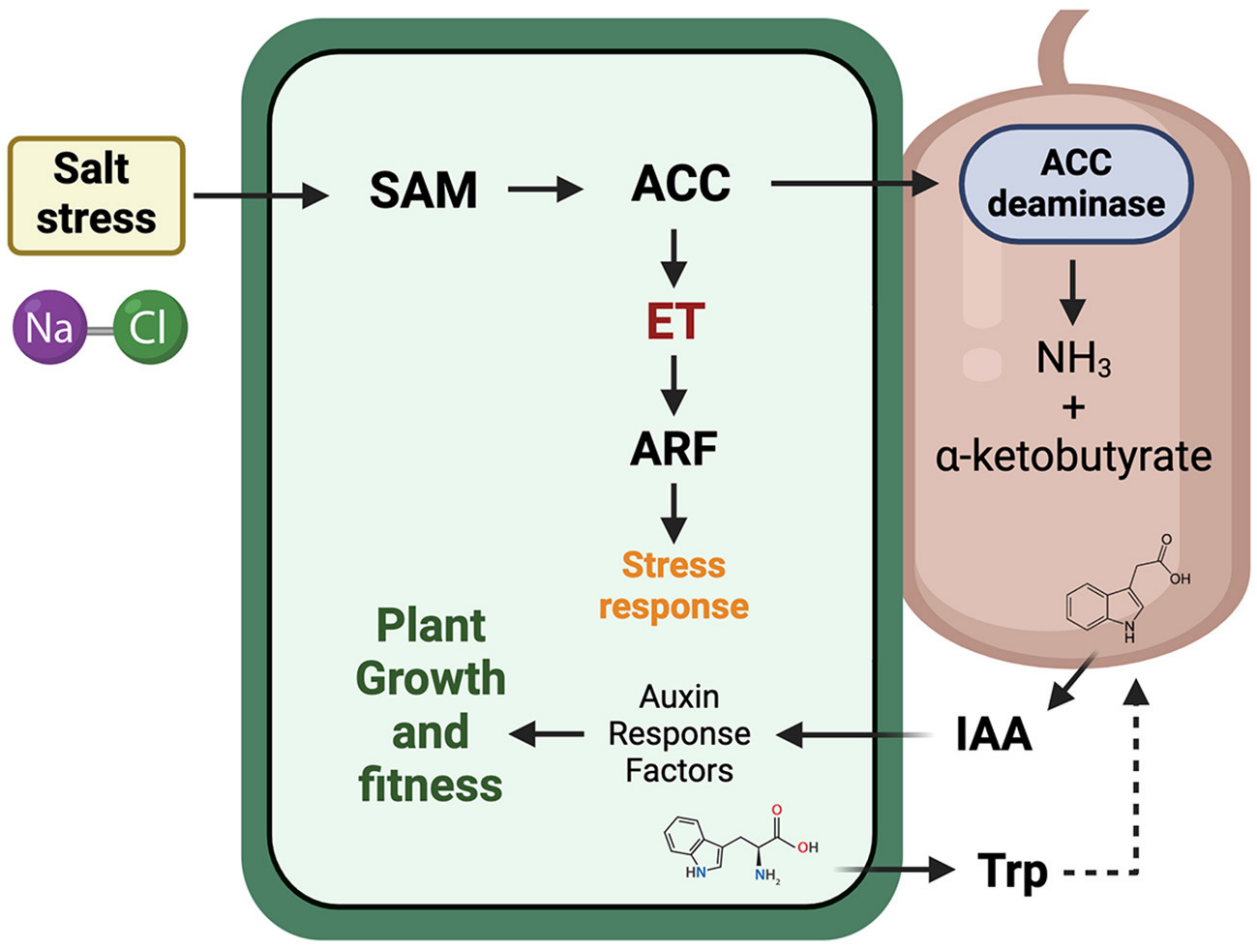
1-aminocyclopropane-1-carboxylic acid (ACC) deaminase model to lower ethylene levels in stressed plants by salinity conditions for the stimulation of plant growth and fitness. Briefly, salt stress induces ethylene synthesis trough the pathway that starts with the production of S-adenosyl-methionine (SAM). Subsequently, SAM is converted to ACC by the enzyme ACC synthase, and in turn, ACC is a direct derivative of ethylene (ET). ACC is then degraded by ACC deaminase enzyme, converting it into α-ketobutyrate and ammonia. This way, the ethylene synthesis is lowering by the degradation of ACC precursor. The model also includes crosstalk with the indole-3-acetic acid (IAA) hormone, where it can be synthesized from plants and/or associated microorganisms.

According to Glick et al. (1998), the ACC produced by the plant can be degraded by the action of microorganisms containing the enzyme ACC deaminase (Figure 1). These microorganisms can inhabit internal tissues or originate from the rhizosphere or narrower sections within the root, such as the rhizoplane. In fact, the exact mechanism by which ACC deaminase-producing microorganisms can detect and recognize ACC is still unknown, although there are numerous reports where inoculations with microorganisms, including fungi or bacteria that promote plant growth, can degrade the plant's ACC. This includes various plant crops of agronomic interest. In this model, ACC is degraded by the action of the ACC deaminase enzyme, converting it into α-ketobutyrate and ammonia. This way, the precursors of ethylene synthesis, i.e., ACC, will be reduced, and consequently, ethylene levels in the plant will decrease, along with all its physiological consequences. Glick's model also includes crosstalk with the indole-3-acetic acid (IAA) hormone and ethylene caused by salt stress. IAA can be synthesized from tryptophan in both plants and associated microorganisms (Etesami and Glick, 2024). Therefore, this hormone, involved in signals that induce growth and cell proliferation in plants through the response to auxin response factors, could stimulate SAM and induce changes in ET concentration. In fact, several studies have demonstrated that microorganisms producing both IAA and ACC deaminase can decrease ET levels in plants and, in turn, stimulate their growth under different stresses, including salinity (Bal et al., 2013; Chandra et al., 2018; Zhang et al., 2019; Murali et al., 2021).

### 4.3 ACC deaminase in *Trichoderma* spp.

The fungi of the genus *Trichoderma* can form associations and establish intimate communication with host plants, even living as endophytes within the plant (Guzmán-Guzmán et al., 2024). Therefore, it is relevant to search for these endophytes to determine their potential functions as growth promoters and contributors to plant health. Such is the case in the study conducted by Wang and coauthors (Wang et al., 2022), where they isolated 59 fungal strains belonging to the genera *Penicillium, Aspergillus, Fusarium, Trichoderma, Rhizopycnis* sp., *Trametes* sp., *Schizophyllum commune* sp., *Alternaria, Cladosporium, Cylindrocarpon*, and *Scytalidium* from the “Seashore mallow” plant (*Kosteletzkya virginica*). *K. virginica* is a non-invasive perennial halophytic oilseed-producing dicot native to the USA Atlantic coasts. ACC deaminase activity was identified in 10 out of the 59 fungal isolates, with two endophytic fungi of the species *T. asperellum* and *T. viride* standing out. These two *Trichoderma* isolates showed the best results when inoculated in wheat (*Triticum aestivum*) and soybean (*Glycine max*), improving various parameters such as plant dry weight and fresh weight, plant height and root length, superoxide dismutase activity, and chlorophyll content. Additional analyses indicated that Trichoderma strains induced a negative regulation of gene expression, including ACC oxidase and ACC synthase, thereby reducing ethylene synthesis and additional damage to plants caused by salinity.

Using two wheat cultivars, one sensitive and another salt-tolerant, a recent work demonstrated that two *Trichoderma* strains (*T. yunnanense* and *T. afroharzianum*) increased salt stress tolerance (along with stimulating proline contents in leaves) compared to non-inoculated plants in both cultivars. Unfortunately, the ability to produce ACC deaminase in these strains was not evaluated, but it was determined that they produced IAA, possibly aiding plant growth through this auxin (Oljira et al., 2020).

In another study using the IAA and ACC deaminase-producing strain *T. longibrachiatum* (TL-6), growth and salt stress tolerance in *Triticum aestivum* plants were increased through various molecular mechanisms. Interestingly, the TL-6 strain regulated the transcription of genes relevant to IAA and ET synthesis, as well as Na+/H+ antiporter genes in both shoots and roots, leading to a decrease in ET synthesis (by diminishing ACC content by up to 22%) and an increase in IAA (approximately 11%), along with a decrease in Na+ accumulation in tissues. The authors suggested that this combination of physiological, biochemical, and molecular responses in wheat plants helped promote growth and salt stress tolerance (Zhang et al., 2019).

Another *Trichoderma* species characterized as beneficial for plants, enhancing their resistance to salt stress (with undetermined ACC deaminase activity), is *T. parareesei*. In this regard, the species *T. parareesei* (and *T. harzianum*), when applied to *Brassica napus* roots, increased productivity and salinity tolerance, as well as drought tolerance, with potentially similar physiological consequences in the plant (Poveda, 2020).

### 4.4 ACC deaminase in *Bacillus* spp.

Within the genus *Bacillus*, there are various species that exhibit high tolerance or resistance to salinity, enabling them to thrive in high concentrations. This makes them perfect candidates as Plant Growth-Promoting Bacteria (PGPBs) and/or bioinoculants in stressful agricultural conditions (Bomle et al., 2021; Ayaz et al., 2022).

In a recent study, Gamalero and Glick (2022) conducted a comprehensive review of the diverse capabilities of various bacteria to ameliorate salinity and drought stress in plants, including several candidates from the *Bacillus* genus. Likewise, other recent review works have also discussed the importance of ACC deaminase (and ethylene) in bacteria, particularly in the *Bacillus* genus, for alleviating salinity stress in multiple crops such as wheat, maize, sorghum, canola, pepper, cucumber, alfalfa, rice, tomato, among others (Etesami et al., 2023). For example, three bacterial strains of the species *Bacillus licheniformis* (RS6569), *Brevibacterium iodinum* (RS16), and *Zhihengliuela alba* (RS111), which possessed ACC deaminase activity, were able to enhance salt stress tolerance in pepper plants (*Capsicum annuum*) by reducing ethylene levels by a 50% less. In addition, the salt tolerance index, that includes the ratio of biomass of salt stressed to non-stressed plants, was also stimulated in inoculated plants with bacilli strains (Siddikee et al., 2011).

Recently, Zhu et al. (2020) evaluated three *Bacillus* strains, namely *B. megaterium* NRCB001, *B. subtilis* subsp. *subtilis* NRCB002, and *B. subtilis* NRCB003, which exhibited ACC deaminase activity, among other mechanisms, on the growth of alfalfa plants under saline stress. In the experiments, two of the strains showed better results (NRCB002 and NRCB003), as they increased the dry weight of the plants compared to non-inoculated seedlings treated with NaCl (130 mM) (Zhu et al., 2020).

Another study demonstrated that two bacilli, *B. marisflavi* (CHR JH 203) and *B. cereus* (BST YS1_42), stimulated defense responses and expression of ROS scavenging and cell rescue genes in pea plants (*Pisum sativum*) subjected to 1% salinity (NaCl) concentrations. Additionally, both strains exhibited high levels of ACC deaminase activity and increased biomass, plant carbohydrates, phenols, flavonoids, and antioxidant enzyme levels in plants, even under salinity stress (Gupta et al., 2021)

There are several studies demonstrating that microorganisms can enhance their beneficial effects on plants when working together as a team (Santoyo et al., 2021a). In other words, a single microbial strain, whether bacteria or fungus, may not possess the complete arsenal of metabolites and activities required to survive and compete efficiently in the soil. Furthermore, in a microbial consortium, one microbe could be synthesizing metabolites inhibiting the growth of phytopathogens while a second one (or third one, etc.) might be stimulating plant growth through direct mechanisms such as hormone synthesis (e.g., IAA, gibberellins, and cytokinins). Therefore, in a microbial consortium, these tasks can be divided and carried out by each microorganism that is part of the consortium, which must be highly compatible and avoid antagonisms among them (Mohanram and Kumar, 2019).

In a recent study, Singh and colleagues (Singh et al., 2023) employed employed a consortium of ACC-deaminase-synthesizing *T. harzianum* (Th) and plant growth-promoting bacteria (PGPB) where synergitic work was observed, including strains Fd-2 (*Achromobacter xylosoxidans*), Ldr-2 (*Bacillus subtilis*), Sd-6 (*Brevibacterium halotolerans*), Art-7 (*Burkholderia cepacia*), and Str-8 (*Halomonas desiderata*), to reduce salt stress in *Ocimum sanctum* (also known as Holy Basil). *O. sanctum* is a medicinal plant in various countries, such as India. The results of this study showed that the coinoculation of Th and PGPB improved plant height and fresh herb weight from 3.78% to 58.76%, with some microbial combinations being more effective than others. Interestingly, coinoculated plants exhibited lower Na+ concentrations and activities of malondialdehyde, H2O2, catalase, and peroxidase. Additionally, plants interacting with microorganisms showed a lower ACC accumulation (49.75% to 72.38% compared to untreated plants subjected to salt stress), suggesting a reduction in the harmful effects caused by salinity stress.

The synergistic action of PGPB and *Trichoderma* functioning as a consortium is crucial to understanding their beneficial capabilities, both in field and greenhouse conditions. Recently, Marghoob et al. (2023) and coauthors evaluated two *Trichoderma* strains (*T. harzianum* and *T. viridae*) and three bacterial strains (*Pantoea* spp. and *Erwinia rhaphontici*) under field conditions and in pot experiments with wheat plants. The results showed that under saline stress, microbial consortia presented encouraging results in various agronomic parameters, such as production, days of flowering and maturity, number of spikes, spike length, spike weight, and number of seeds per spike, among others. Although the microorganisms did not produce the auxin IAA, they exhibited ACC deaminase activity, among other mechanisms relevant to promoting plant growth, such as the production of siderophores, antioxidant activities, and exopolysaccharide/biofilm production.

Another recent study evaluated the synergistic action of *Trichoderma koningiopsis* NBRI-PR5 (MTCC 25372) and *T. asperellum* NBRI-K14 (MTCC 25373), named TrichoMix, in rice crops under saline stress conditions. The results concluded that this consortium increased the accumulation of osmoprotectors in rice plants, modulated the plant's defense system, and improved grain production. Additionally, rice grains showed an increase in nutrients such as Fe and Zn by approximately 40% and 29%, respectively. Together, these studies demonstrate that various species and strains of *Trichoderma* can be an excellent option to stimulate the growth and production of various crop plants and may not only exhibit mycoparasitic action, as commonly known for this genus of biocontrol fungi (Anshu et al., 2022).

## 5 Stimulation of plant growth and health under salt stress

Under saline conditions, plants activate multiple physiological and biochemical mechanisms that are responsible for adaptation to hostile environments. Furthermore, plants interact with beneficial soil microbes to minimize the negative effects of salt stress. Plants recruit beneficial microorganisms under stressful growing conditions via the root exudation of metabolites (Shrivastava and Kumar, 2015; Gupta et al., 2022). For example, tomato root (*Solanum lycopersicum*) exudate oxylipins are chemoattractants of the beneficial fungus *Trichoderma harzianum* under saline stress (100 mM NaCl), causing significant changes in the reorientation of germ tubes (Lombardi et al., 2018). Moreover, organic acids, such as stearic and palmitic acids, released by the halophyte *Limonium sinense* under saline conditions favor motility and chemotaxis in the strain *Bacillus flexus* KLBMP 4941 (Xiong et al., 2020).

**Figure 2:**
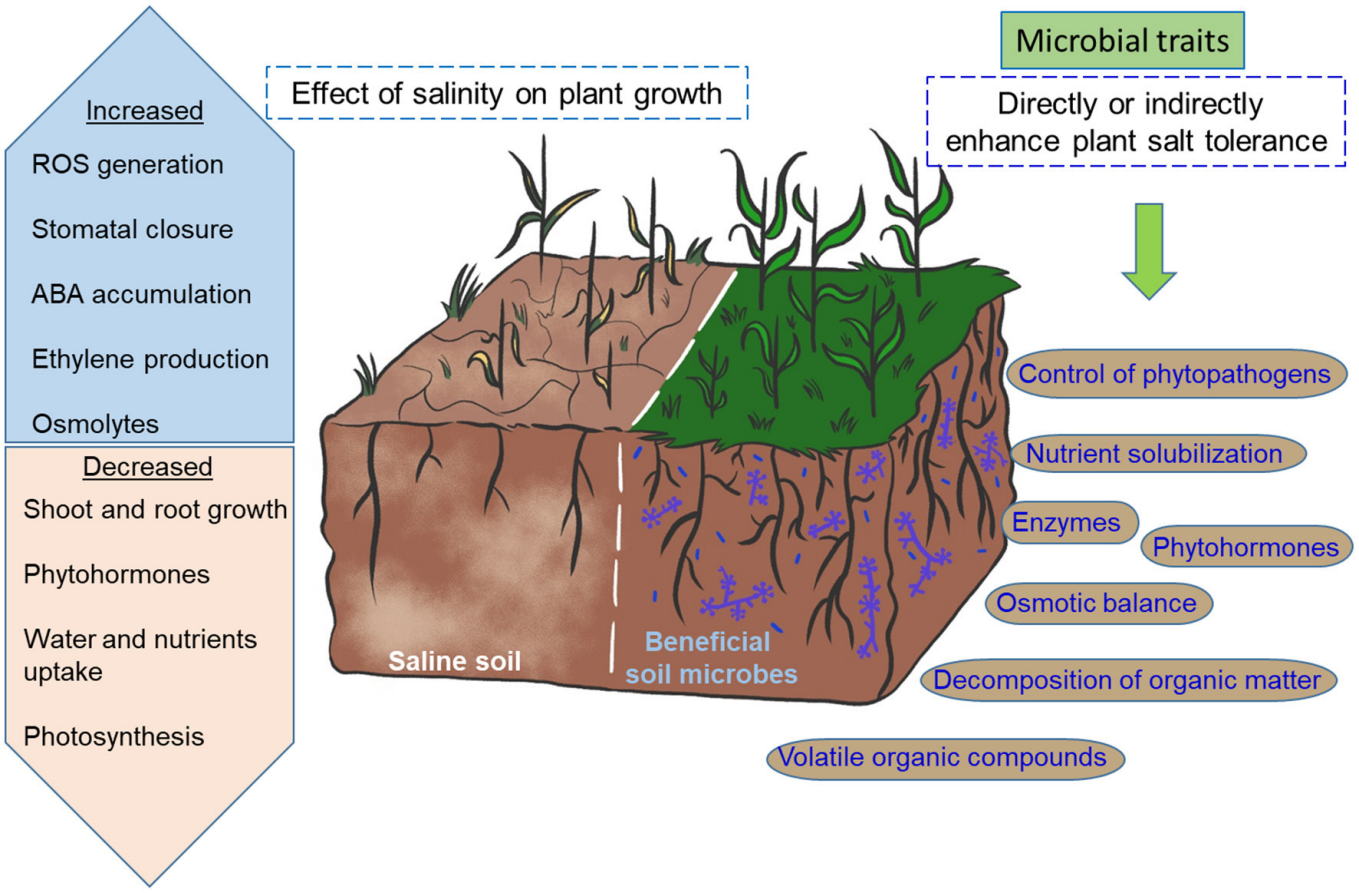
Activation of salt stress tolerance mechanisms in plants by plant growth-promoting microorganisms. The metabolic processes, gene expression, and physiological responses to the environment are severely affected when plants are under salinity conditions. Beneficial microorganisms produce phytohormones (e.g., indole-3-acetic acid) and volatile organic compounds that modulate plant growth and defense under saline conditions. In particular, the root system is strengthened, facilitating the uptake of water and nutrients from the soil. In contrast, beneficial microorganisms induce the accumulation of abscisic acid in plants, which participates in regulating stomatal closure and controlling water loss. Likewise, they increase the antioxidant systems by improving the enzymatic antioxidant activities (e.g., SOD) and promoting the accumulation of osmolytes (e.g., proline) and antioxidants (e.g., ascorbic acid) in plants. Alternatively, microbes decrease the excessive production of ethylene by producing 1-aminocyclopropane-1-carboxylic acid (ACC)-deaminase and induce the elimination of the Na+ ion through root exudates. These effects combined can contribute to the adaptation and survival of the plants to salt stress.

The intertwined action of beneficial soil microbes occurs at three proposed levels: (1) the survival of microorganisms themselves in a hyperosmotic environment; therefore, the use of halotolerant bacterial or fungal isolates seems to be an excellent choice; (2) the induction of salt-tolerant mechanisms in plants; and (3) the improvement of soil quality (Figure 2). These microbial actions cannot be performed without the production of secondary metabolites known as specialized metabolites (Fouillaud and Dufossé, 2022). These compounds are structurally diverse, and their production is influenced by nutrient sources, growth stages, and environmental conditions. Moreover, fingerprints of microbial compounds are species-specific and play multifunctional roles in plants (Jalali et al., 2017). Several studies on the effect of *Trichoderma* spp., *Bacillus* spp., or their metabolites on ameliorating the negative effects of salt stress in plants and structuring agroecosystems are continually emerging. New halotolerant strains have been isolated from different environments, and their potential as plant growth promoters under saline conditions could improve crop growth and productivity (Ibarra-Villarreal et al., 2021; Prabhukarthikeyan et al., 2022; Zhang et al., 2022).

**Figure 3:**
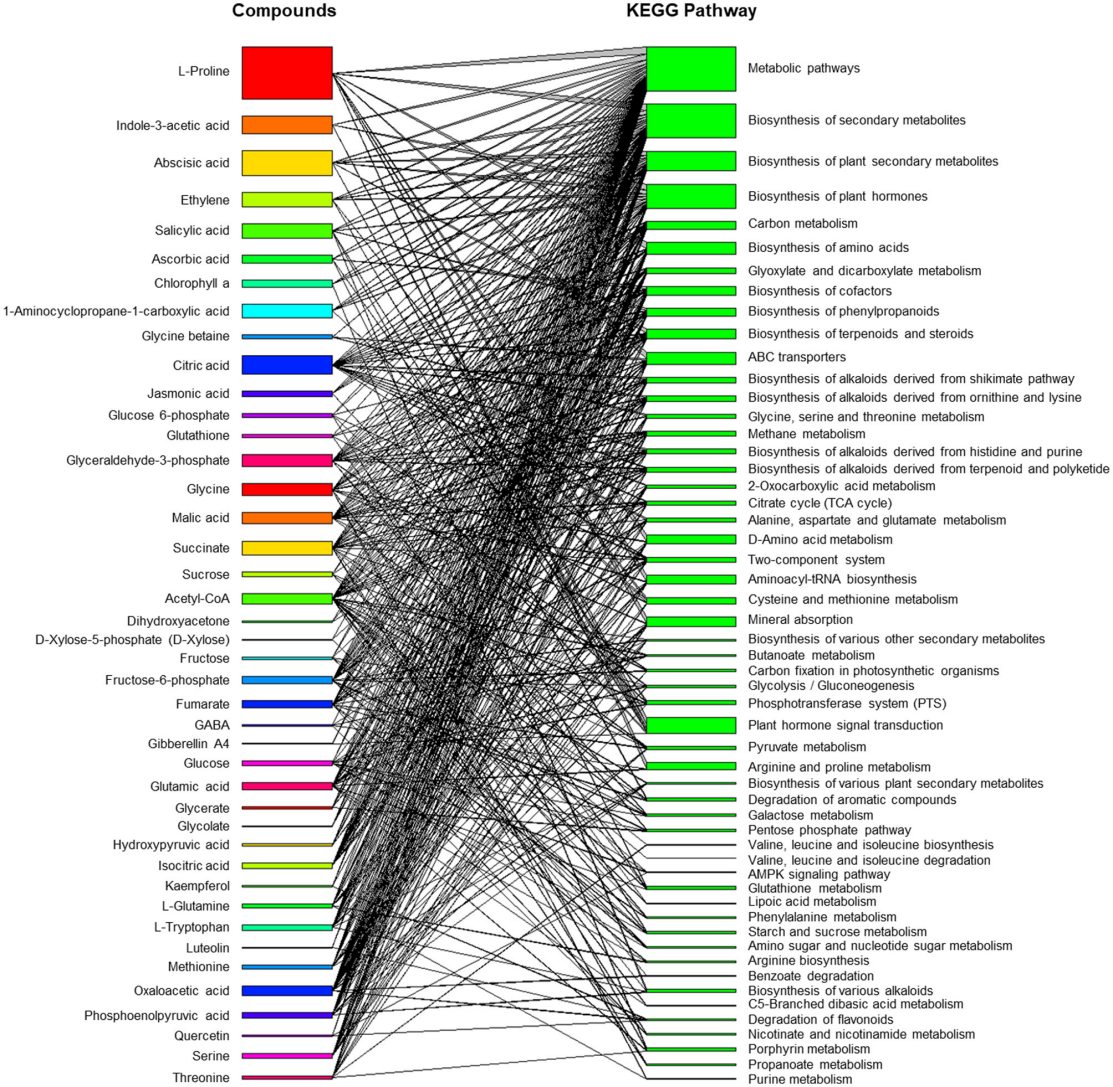
Bipartite network of metabolite-metabolic pathway connections involved in plants in response to the inoculation of *Trichoderma* spp. and *Bacillus* spp. under saline conditions. The bipartite interaction network was built from published literature and by using KEGG Pathway Database entries. Full data was available in a public repository (https://github.com/artur14785/salinity_bipartite).

*Trichoderma* and *Bacillus* can induce existing metabolic machinery in plants to cope with saline stress (Figure 3). The bipartite network displays a list of metabolites produced by plants grown under saline conditions and during interactions with these beneficial soil microorganisms and how these compounds are connected to the Kyoto Encyclopedia of Genes and Genomes (KEGG) metabolic pathways (Ogata et al., 1999). The network showed that both primary and secondary metabolisms were finely modulated to enable a physiological response in plants to overcome stress. The induction of osmolyte synthesis is one of the mechanisms triggered by microbes, and proline is the most studied because its accumulation is highly correlated with salt tolerance. Therefore, it is overrepresented in the network and connected to the biosynthesis of other metabolites and ABC transporters. Proline is a precursor of proteins that play important roles in plant growth and differentiation across the life (Kishor et al., 2015). The role of microbes in phytohormone homeostasis, such as indole-3-acetic acid (IAA), abscisic acid (ABA), salicylic acid (SA), and jasmonic acid (JA), has also been well reported (Contreras-Cornejo et al., 2009; Ueda et al., 2012; Chen et al., 2020). These phytohormones are associated with plant hormone signal transduction or the biosynthesis of metabolites, such as those involved in defense or tolerance to stressful growing conditions. Furthermore, the modulation of carbon metabolism and the promotion of the synthesis of secondary metabolites, such as flavonoids, indicate how complex and multivariate plant responses can occur during plant and microbe interactions under control and saline conditions (Ullah et al., 2019; Zulfiqar et al., 2019). Therefore, deciphering the molecular mechanisms underlying these interactions is challenging.

### 5.1 Plant growth promotion by *Trichoderma* spp.

*Trichoderma* spp. are free-living fungi that are commonly found in soil and root ecosystems. Approximately 375 species have been described worldwide (Cai and Druzhinina, 2021). Some *Trichoderma* species are more sensitive to salt stress than others, which restricts fungal growth and spore production (Zhang et al., 2019; Yusnawan et al., 2021; Abdelrhim et al., 2024). *Trichoderma* is widely known for its ability to produce antibiotics, parasitize other fungi, and to compete with phytopathogens. Together, these indirect mechanisms are considered the basis of *Trichoderma* to exert beneficial effects on plant growth and development; in fact, these abilities are maintained under salt conditions. For example, the average inhibition rates of *T. longibrachiatum* TG1 against *Fusarium pseudograminearum* were 33.86% and 46.62% at 0 and 150 mM NaCl, respectively. The antifungal effect correlated with the increased relative expression of enzymes, such as chitinases and glucanases, involved in the lysis of fungal host cells. In addition, TG1 induced salt tolerance in wheat seedlings (*Triticum aestivum*) and reduced the incidence of disease caused by *F*. *pseudograminearum* (Boamah et al., 2021), indicating the effectiveness of *Trichoderma* in mitigating combined stress. Similarly, plant growth promotion and biocontrol effects have been reported for different isolates of *Trichoderma* under salinity (Sánchez-Montesinos et al., 2019; Ding et al., 2021; Liu et al., 2023).

Alternatively, some *Trichoderma* spp. directly influence plants by producing growth-regulating substances such as IAA and ethylene, activating defense responses dependent on JA, SA, and/or ethylene, and inducing changes at the biochemical level to confer adaptation to abiotic stress (Contreras-Cornejo et al., 2009; Macías-Rodríguez et al., 2020). *T. virens* produces the auxin IAA (13.48 g L^−1^), as well as other auxin related compounds such as indole-3-ethanol (72.33 g L^−1^) and indole-3-acetaldehyde (59.40 g L^−1^) from tryptophan in potato dextrose broth (PDB) culture medium. IAA production varies among fungal species and strains (Kumar and Verma, 2018). Importantly, the exogenous addition of salt (100 mM NaCl) to PDB culture medium did not affect the production of auxin-like compounds (Contreras-Cornejo et al., 2015). Similarly, halotolerant species can produce IAA, indicating their potential for use in sustainable agriculture (Kumar et al., 2017; Oljira et al., 2020). Auxins are phytohormones that regulate various processes that are involved in plant growth and development. Salt undoubtedly affects plant growth and development in a dose-dependent manner and interrupts the root system architecture (primary root growth and the formation of lateral and adventitious roots), with repercussions on nutrient and water uptake and anchoring to the soil (Steffens and Rasmussen, 2016). An agravitropic response called “halotropism” is induced at the root. Positive or negative halotropic movements depend on the type of plant, salt gradient in the soil, and exposure time to saline stress. The directional response of roots to salt environments has been related to the redistribution of auxins, indicating an important role of auxin-dependent mechanisms in plant adaptive responses (Szepesi, 2020). Therefore, it is reasonable to speculate that auxins released by *Trichoderma* may be important for maintaining root development under salt stress. This is the case for *T. atroviride* and *T. virens*, which alter root system architecture and auxin-inducible gene expression in *Arabidopsis* during fungal colonization (Contreras-Cornejo et al., 2009). In addition, *Trichoderma* can regulate endogenous ACC in plants by producing ACC deaminase, which diminishes the deleterious increase in ethylene levels and stimulates the root elongation under salt stress, an event that does not occur when plants are inoculated with ACC deaminase-silenced mutants. Furthermore, ACC deaminase production by certain *Trichoderma* species, such as *T. longibrachiatum* T6, under NaCl stress enhanced the growth and tolerance of wheat plants to abiotic stress (Zhang et al., 2019).

The volatile organic compounds (VOCs) emitted by *Trichoderma* act as signals that regulate growth and defense programs in several plant species. In particular, *Arabidopsis* perception of 6-pentyl-2*H*-pyran-2-one (6-PP) produced by *T. atroviride* involves components of auxin transport and the ethylene response regulator EIN2, which orchestrates fundamental processes during root system architecture configuration (Garnica-Vergara et al., 2016). 6-PP is also produced by other *Trichoderma* spp. and exhibits plant growth-promoting properties (Lee et al., 2016). However, the pool of fungal VOCs may exert a varied response in plants compared with the exogenous application of 6-PP. Other fungal VOCs reported in *Trichoderma* belong to the family of alcohols, ketones, and terpenes and elicit both developmental and defense programs in plants (Yusnawan et al., 2021). However, much work is required to fully understand the mechanisms triggered by these molecules during plant-microbe interactions. VOCs produced by *Trichoderma* under salt stress induce salt tolerance mechanisms in plants. For example, VOCs from *T. koningii* at 100 mM NaCl diminished H_2_O_2_ accumulation in *Arabidopsis* plants compared with uninoculated plants, inducing protection against oxidative damage (Jalali et al., 2017).

*Trichoderma* can produce ABA with and without a salt stimulus. This phytohormone is involved in the regulation of abiotic stress tolerance. The species *T. atroviride* and *T. virens* are salt tolerant at 100 mM NaCl, and they slightly increase ABA production (from 0.11 to 0.33 ng mL^−1^ for *T. virens* and 0.15 to 0.21 ng mL^−1^ for *T. atroviride*) when grown in PDB culture media supplemented with 100 mM NaCl. Hence, an accumulation in the shoots regulating stomatal opening through a mechanism dependent on the phosphatases ABI1 and ABI2 to avoid water loss was observed in plants during fungal interactions with *Arabidopsis* (Contreras-Cornejo et al., 2015).

Plants inoculated with *Trichoderma* have shown increased expression of many genes involved in osmoprotection and general oxidative stress in shoots and roots (Abdelrhim et al., 2024). For instance, fungi improve the enzymatic antioxidant activities of SOD, POD, and CAT, among others, in plants as scavenging mechanisms to mitigate ROS damage. In addition, *Trichoderma* spp. increase the elimination of Na+ through root exudates as part of a detoxification mechanism in plants under salt stress, and some studies have reported an accumulation of proline as an osmolyte and ascorbic acid as an antioxidant agent to detoxify the cell from ROS, especially H_2_O_2_. Similarity, Zhang et al. (2019) observed an increased content of proline, ascorbate, and glutathione, and decreased levels of H_2_O_2_ in wheat seedlings (*Triticum aestivum*) inoculated with *T. longibrachiatum* grown under saline conditions. The downregulation of proline production in colonized plants induced by *Trichoderma* suggests a complex role for proline in ameliorating saline stress and its interaction with beneficial soil microbes (Oljira et al., 2020).

Studies carried out in the field indicate that inoculation of *Trichoderma* in stressed plants grown under saline conditions modulates nutrient uptake and bacterial community structure and function in the rhizosphere soil. This is the case for *T. harzianum* ST02, which promotes the growth of sweet sorghum (*Sorghum bicolor*), facilitates nutrient uptake from saline soil, and modulates the relative abundance of Actinobacteria in the rhizosphere (Wei et al., 2023).

### 5.2 Plant growth promotion by *Bacillus* spp.

*Bacillus* has been widely studied because of its capacity to survive under different environmental conditions and its ability to produce a wide spectrum of metabolites for medical, industrial, and agricultural applications (Santoyo et al., 2012; Valenzuela-Aragon et al., 2019). In particular, this genus has gained great interest as a promoter of the health and performance of crops grown in non-saline and saline soils by inducing metabolic and molecular reprogramming in plants to support growth and stress control (Abd El-Daim et al., 2019; Etesami et al., 2023) such as *B. subtilis, B. subtilis, B. amyloliquefaciens, B. licheniformis*, and *B. pumilus* (Caulier et al., 2019). Salt tolerance varies between species and strains. Salt may affect bacterial growth, biofilm formation, secondary metabolite production, and exoproteome profile, which are important bacterial traits for salt stress tolerance and competitive capacity in the rhizosphere (Massawe et al., 2018; Ullah et al., 2019; Ibarra-Villarreal et al., 2021; Santoyo et al., 2021b; Singh et al., 2022). However, the synthesis of certain metabolites is induced, favoring their interaction with plants, such as glycine betaine and ectoine, which are produced for cellular adaptation to high-osmolarity environments but also play a role as osmoprotectors in plants (Asaf et al., 2017; Zamanzadeh-Nasrabadi et al., 2023; Valencia-Marin et al., 2024).

The ability to form endospores and produce antimicrobial compounds is advantageous for bacterial survival in saline soils, where the carbon source is scarce and competition for space and nutrients prevails (Caulier et al., 2019). Some antimicrobial compounds include non-ribosomally synthesized peptides, lipopeptides, polyketide compounds, bacteriocins, and siderophores, which are structurally diverse among *Bacillus* spp. (Rodríguez et al., 2018; Blake et al., 2021; Etesami et al., 2023). In addition, *Bacillus* produces HCN and extracellular hydrolytic enzymes including cellulase, protease, and xylanase, which play important roles in the lysis of pathogen cell walls (Martínez-Absalón et al., 2014; Bhagwat et al., 2019). Importantly, the antagonistic activity of *Bacillus* spp. modifies the rhizosphere microbial community, reducing the presence of phytopathogens and indirectly influencing plant growth. Plants are usually more susceptible to phytopathogen attack under saline conditions (Cramer et al., 2011; Vanderstraeten et al., 2019; Zhou et al., 2021; Patel et al., 2023). It is possible to study the sensitivity of phytopathogens to antimicrobial compounds produced by *Bacillus* and how they are differentially produced according to the phytopathogen tested using confrontation assays (Cossus et al., 2021). Additionally, *in planta* assays have revealed the potential of *Bacillus* spp. in biocontrol (Chowdhury et al., 2015; Maan et al., 2022).

*Bacillus* directly ameliorates salinity stress in plants through multiple mechanisms that involve the production of phytohormones, VOCs, and ACC deaminase, which decreases ethylene levels, osmoprotectants, and exopolysaccharides (Panneerselvam et al., 2019; Etesami et al., 2023). Regarding phytohormones, *Bacillus* produces IAA in nutrient broth through the L-tryptophan pathway, and salt stress differentially affects IAA production (Duca et al., 2014; Etesami and Maheshwari, 2018). Furthermore, modulation of IAA homeostasis is observed during bacterial association with plants, resulting in the stimulation of cell elongation and promotion of plant growth (Kumar et al., 2021). Plant responses are specific to the bacterial species or strain, even more so when an external stress stimulus, such as salt stress, affects bacterial growth and colonization (Zakavi et al., 2022). Other phytohormones that can be produced by *Bacillus* spp. under saline and non-saline conditions are gibberellins (GAs), cytokinins, and ABA, which can presumably be taken up by plants to regulate endogenous phytohormone content and improve plant physiology under saline and non-saline conditions (Ilangumaran and Smith, 2017; Hedden, 2020; Glick, 2023). For example, *B. amyloliquefaciens* RWL-1 and *B. methylotrophicus* KE2 produce gibberellins, including GA_4_, which is the most bioactive in plants, increasing seed germination and subsequent plant development in *Lactuca sativa, Cucumis melo, Glycine max, Brassica juncea*, and *Oryza sativa* (Radhakrishnan and Lee, 2016; Keswani et al., 2022). Another study reported that RWL-1 also produces ABA, thereby increasing its potential for salinity stress tolerance (Shahzad et al., 2016). Other species, such as *B. marisflavi* produce xanthoxin-like compounds (ABA analogs) that benefit the physiological response of *Brassica juncea* to drought stress (Gowtham et al., 2020). *B. subtilis* produces different cytokinins that benefit the growth and development of *Triticum durum* and *Lactuca sativa* (Arkhipova et al., 2005, 2007). Indeed, modulation of endogenous phytohormone levels by *Bacillus* spp. upon exposure of plants to salinity stress has been widely reported. In particular, *Bacillus* upregulates ABA synthesis- and sensing-related genes in plants (Yoo et al., 2019; Galicia-Campos et al., 2023). ABA plays an important role in the adaptive response to abiotic stress; thus, ABA content increases in inoculated plants, similar to osmoprotectant compounds (i.e., proline, polyamines, glutamate, and total free amino acids) and antioxidants (i.e., glutathione, ascorbic acid, flavonoids, and phenols). The activities of antioxidant enzymes such as POD, CAT, and SOD were improved, indicating that *Bacillus* helps plants neutralize high levels of ROS and reduce lipid peroxidation, rescuing them from salt stress (Egamberdieva et al., 2017; Ullah et al., 2019; Galicia-Campos et al., 2023).

*Bacillus* spp. are known to emit bioactive VOCs that modulate plant endogenous hormones, nutrient uptake by roots, and induction of systemic defenses in plants (Choudhary and Johri, 2009; Zhou et al., 2021). Ryu et al. (2004) and Sharifi and Ryu (2018) were pioneers in investigated the beneficial effects of microbial VOCs on plant growth. The authors analyzed the VOCs profiles emitted by *B. subtilis* GB03 and *B. amyloliquefaciens* IN937a and found that acetoin and 2,3-butanediol enhanced the growth of *Arabidopsis* and modulated the severity of infection caused by the phytopathogen *Erwinia carotovora*. Moreover, VOCs produced by GB03 triggered the induction of a high-affinity K+ transporter (HKT1) in shoots and reduction of HKT1 in roots, limiting Na+ entry into the roots and facilitating shoot-to-root Na+ recirculation in *Arabidopsis* plants exposed to saline stress (100 mM NaCl), indicating that bacterial VOCs diminish the adverse effects caused by salt. Similarly, Liu et al. (2014, 2023) reported that VOCs from *B. amyloliquefaciens* FZB42 increased POD, CAT, and SOD activity and decreased Na+ content in stressed plants. In addition, transcriptomic profiling of genes from *Arabidopsis* shoot tissues has provided insights into other molecular mechanisms responsible for inducing salt tolerance via bacterial VOCs emissions. The beneficial effects of the VOCs emitted by *Bacillus* spp. have been explored in plants grown under saline conditions. These compounds promote an increase in the levels of phytohormones, such as JA, SA, and osmolytes, as well as nutrient uptake from the soil (Holopainen and Gershenzon, 2010; Jalali et al., 2017; Cappellari and Banchio, 2020). *Bacillus* spp. produces acetoin and four-carbon alcohols. However, the overall bacterial VOCs profiles differed among them; consequently, the *Arabidopsis* seedling response was in accordance with the perceived bacterial chemical signature (Gutiérrez-Luna et al., 2010).

*Bacillus* spp. display different abilities to increase the uptake and accumulation of plant nutrients via different mechanisms, such as phosphate solubilization, siderophore production, and nitrogen fixation, which contribute significantly to enhanced plant growth under saline conditions. Salt-tolerant species can better achieve these bacterial traits because they can survive in osmotic and ionic environments (Kanekar et al., 2012; Ibarra-Villarreal et al., 2021; Lahsini et al., 2022; Zamanzadeh-Nasrabadi et al., 2023). In addition, certain members of the *Bacillus* genus produce exopolysaccharides that aid bacterial colonization, improve soil aggregation, and enhance the uptake of water and nutrients. The monosaccharide composition of this polymer changes in the presence of stress. Furthermore, exopolysaccharides bind to toxic Na+, restrict Na+ influx into roots, and promote water retention capacity through biofilm formation on root surfaces (Singh et al., 2022; Valencia-Marin et al., 2024). See Table 1 for a list of recent examples of beneficial activities exerted by *Trichoderma* and *Bacillus* spp. on crops under saline conditions.

**Table 1:** Examples of *Trichoderma* and *Bacillus* species (and other amendments) beneficiating plant crops under salt stress.

**Microbial species**	**Beneficiated host plant (Species)**	**Plant growth-promoting activities under salt stress**	**References**
*Trichoderma koningiopsis and Trichoderma asperellum*	Rice (*Oryza sativa*)	Increased plants' osmoprotectors and defense system. Improved grain production	Anshu et al., 2022
*Trichoderma harzianum and biochar*	Spinach (*Spinacia oleracea* L.)	Improved plant growth parameters, chlorophyll content, mineral contents and the levels of endogenous phytohormones	Sofy et al., 2022
*Trichoderma longibrachiatum*	Wheat (*Triticum aestivum* L.)	Improved resistance to pathogens attack (Fusarium pseudograminearum) and increased the superoxide dismutase (SOD), peroxidase (POD), and catalase (CAT) activities	Boamah et al., 2021
*Trichoderma yunnanense, Trichoderma afroharzianum*, and *Bacillus licheniformis*	Wheat (*Triticum aestivum* L.)	Enhanced net photosynthesis, water use efficiency, and growth of wheat plants	Oljira et al., 2020
*Trichoderma harzianum, Achromobacter xylosoxidans, Bacillus subtilis), Brevibacterium halotolerans, Burkholderia cepacia, and Halomonas desiderata*	*Holi Basil (Ocimum sanctum)*	Lower concentrations of Na+ and activities of malondialdehyde, H2O2, catalase, and peroxidase. Lower accumulation of ACC	Singh et al., 2023
*Trichoderma atroviride*	Cucumber (*Cucumis sativus* L.)	Ameliorated salt stress and diminishes cucumber root rot caused by the phytopathogen *Fusarium oxysporum*	Zhang et al., 2022
*Bacillus subtilis* and *Pseudomonas fluorescence*	Common bean (*Phaseolus vulgaris*)	Augmentin plant growtha and yield. Some biochemical activities	Kumar V. et al., 2020
*Bacillus subtilis* and *AMF*	Sulla (*Sulla carnosa*)	Increment in plant growth and nutrition (N, P, K, Mg, Cu, and Fe)	Hidri et al., 2019
*Bacillus firmus*	Alfalfa (*Glycine max*)	Better performance and plant growth increment	El-Esawi et al., 2018
*Bacillus paralicheniformis*	wheat (*Triticum turgidum* subsp. *durum*)	Mitigation of the negative effects of saline soil on wheat	Ibarra-Villarreal et al., 2021
*Brevibacterium epidermidis and Bacillus aryabhattai*	Canola (*Brassica campestris* L.)	Increment in amylase, invertase, and protease activities, and decreased ethylene levels	Siddikee et al., 2015
*Brevibacterium iodinum, Bacillus licheniformis, and Zhihengliuela alba*	Pepper (*Capsicum annuum*)	Lower ethylene production, plant biomass increment	Siddikee et al., 2011
*Bacillus megaterium, Bacillus subtilis subsp. subtilis, and Bacillus subtilis*	Alfalafa (*Medicago sativa* L.)	Increased dry weight	Zhu et al., 2020
*Bacillus tequilensis*	Rice (*Oryza sativa*)	Improved biochemical attributes and nutrient accumulation	Shultana et al., 2021
*Bacillus marisflavi and Bacillus cereus*	Pea (*Pisum sativum*)	Improved plant biomass, increment in plant carbohydrates and antioxidant enzymes levels. Upregulation of plant ROS scavenging and defense genes	Gupta et al., 2021

## 6 Microbial-assisted phytoremediation processes of salt-affected soils

Usually, agricultural crops are not tolerant to soil salinity, so one strategy is to develop new salt-tolerant varieties. Nevertheless, some plant species have failed to realize their potential for growth, development, and production in saline soils. In addition to salinity stress, which can affect more than 75 countries worldwide, soils containing high amounts of Na, Mg, Cl, and Ca, among other elements, are found in arid or semi-arid regions, leading to additional stress in plants (Munns and Gilliham, 2015).

**Figure 4:**
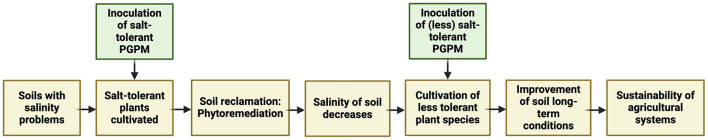
The pathway for saline soil phytoremediation assisted by halotolerant microbes, such as *Trichoderma* and *Bacillus* species, conducting research toward a sustainable production in agriculture.

The fertility and overall health of saline (and sodic) soils can be remediated through processes such as phytoremediation. Phytoremediation involves the use of plant species (some of which are trees) that can tolerate and accumulate high concentrations of salt (hyperaccumulators and halophytes). In turn, they decrease the salt concentration in the soil. Phytoremediation also involves calcium nutrition in the soil, resulting in the removal of sodium through cation exchange (Ashraf et al., 2010). Plant species, such as *Tamarix smyrnensis, Brassica* spp., *Portulaca oleracea, Chenopodium album*, and *Glycyrrhiza glabra* have been employed in soil remediation processes to address salinity issues. Given the well-established fact that each plant analyzed for its association with soil microorganisms and endophytes prompts the question of whether these can stimulate plant mechanisms to reclaim saline soils worldwide. Figure 4 illustrates a scheme adapted from the work of Imadi et al. (2016), depicting the pathway for saline soil phytoremediation, assisted by a halotolerant microbiome. The ultimate goal is to improve the fertility of agricultural ecosystems with soil problems and to achieve sustainability in food production.

The plant-associated microbiome is the second genome that can enhance tolerance to salt stress, among other types of abiotic and biotic stresses. Therefore, it is expected that there is synergy between halophilic microorganisms with ACC deaminase activity and plants, some of which are phytoremediators. For example, Chang et al. (2014) demonstrated that plant growth-promoting bacteria (PGPBs) of the genus *Pseudomonas* with ACC deaminase activity isolated from a salt-impacted (~50 dS/m) farm field promoted the growth of barley and oats in soil under salt stress (1% NaCl) in greenhouse (9.4 dS/m) and field trials (6–24 dS/m). The most notable result was that PGPB treatment stimulated greater plant biomass, leading to increased salt absorption and a reduction in soil salinity.

In a microcosm experiment, Anees et al. (2020) studied the effectiveness of phytoremediation combined with the stimulating role of bacteria (with salt tolerance of up to 20% in culture media) for the reclamation of saline soils. Interestingly, after the analysis, it was observed that the soil salinity was reduced by bacterial action from 6.5 to 2 dS/m. Other factors, such as the weight of both fresh and dry shoots and roots, increased with bacterial inoculation compared to the control in saline soils, and the Na/K ratio decreased in the plant tissues.

The issue of salinity can be exacerbated by contamination with other metals such as nickel (Ni), as reported by Ma et al. (2019) in a recent study. In this study, it was observed that a group of soil microorganisms, including the salt-resistant and plant-beneficial bacterium *Pseudomonas libanensis* strain TR1 and the arbuscular mycorrhizal fungus (AMF) *Claroideoglomus claroideum* strain BEG210, improved the saline conditions of soil in interaction with the plant Helianthus annuus. The results specifically showed that *C. claroideum* significantly improved plant growth, altered the physiological state, as well as the potential for Ni and sodium (Na+) accumulation by H. annuus under Ni and salt stress, either separately or in combination.

## 7 *Trichoderma-Bacillus* synergisms and consortia

Understanding the interactions that occur between microorganisms, in addition to those that occur with their plant hosts, is crucial for designing effective bioinoculants in field situations. In open fields, environmental conditions can be unpredictable, with events such as floods, droughts, strong winds, pests, and other factors often challenging an inoculant's ability to promote plant growth and increase production, especially under saline conditions. For *Trichoderma* and *Bacillus*, we have provided several examples of synergisms between these soil microorganisms that benefit plant crops. However, we propose a series of analyses that should be evaluated prior to designing bioinoculants based on *Trichoderma* and *Bacillus*.

### 7.1 Individual effects

Before determining if two microorganisms can function as a consortium to promote plant growth, their individual interactions should be evaluated. It seems obvious that each microbial strain should have a beneficial effect, but in various studies, this treatment is omitted, focusing only on the consortium activity, leaving uncertainty about the individual effect of each microorganism. Sometimes, multi-species consortia have more than three or four groups of strains, but even so, there should be an evaluation of each one to determine if its participation should be omitted. This would help facilitate the production of a consortium that contains only the microorganisms that truly have an active role in improving crop quality.

### 7.2 Evaluate strain compatibility

Strain compatibility is another important aspect to evaluate to avoid certain antagonisms between each species or strain in a microbial consortium. Each interaction between each species or strain in the consortium should be evaluated individually. Of course, the more microbial groups a consortium contains, the harder it will be to determine the compatibility of all strains. This situation becomes complicated in the case of microbial transplants that may contain hundreds or thousands of phylogenetically distinct groups and still have a beneficial effect on crops. However, as much as possible, it is advisable to know the individual effect of each participant in a microbial consortium.

### 7.3 Determine dual or synergistic effects of action mechanisms

Once individual effects are known, it is important to understand the group behavior of each strain. Some microbial groups like *Bacillus* or *Streptomyces* are biofactories of compounds that are excreted into the external environment and affect their “consortium partners.” In a recent study, Guzmán-Guzmán et al. (2024) evaluated the interaction between strains of PGPRs (*Bacillus, Rouxiella*, and *Pseudomonas*) and *Trichoderma*, determining that some, like *Rouxiella*, perform better at inhibiting the growth of pathogens like *Fusarium* spp. when co-inoculated with *Trichoderma*. On the other hand, a strain of *Pseudomonas* showed better interaction with the same strain of Trichoderma to stimulate the growth of *Arabidopsis* plants. Finally, a strain of *Bacillus* was found to be a good plant growth stimulator, but when interacting with Trichoderma, it inhibited its growth, so it was determined that its action was more beneficial when acting individually rather than in co-inoculation with Trichoderma.

### 7.4 Determine the tripartite interaction plant-pathogen-inoculant

Having multiple participants in a consortium is complicated; determining their beneficial role can be even more unpredictable when adding a new participant, such as one or more pathogens that can be fungi, viruses, nematodes, or bacteria, among others. One of the mechanisms to evaluate in these interactions, besides the direct action to inhibit the growth of potential pathogens, is the stimulation of the plant's immune system (or ISR). In some cases, one or more strains may directly antagonize the pathogen, while another strain might strengthen the plant by stimulating ISR (Choudhary and Johri, 2009). This type of function complementation is highly desirable in a consortium, as each participant can benefit their plant host through multiple mechanisms without competing with each other.

### 7.5 What is the effect of the consortium on the host's endemic microbiota?

At the beginning of this subheading, we mentioned understanding the multiple interactions between the microbial participants of a bioinoculant. In this case, it is also advisable to know if the consortium will impact the plant's endemic microbiome, particularly the microbiota that also has beneficial effects on its host. There are several reports where the inoculation of bacterial groups can affect beneficial microbial groups, but there are also data where the inoculation of bacteria like Bacillus can have beneficial effects and stimulate the abundance of other microbial populations that also benefit crops. In some cases, they stimulate groups like AMF, which also improve soil health, contributing to an integral impact on the agroecosystem.

## 8 Future challenges

In the previous paragraphs, we suggested some recommendations to improve the behavior of synergisms in microbial consortia, not only of *Trichoderma-Bacillus* but also in general for microbial consortia as bioinoculants, whether they act as fertilizers, antagonists, or biostimulants. Therefore, they are not the only factors for achieving the success of a bioinoculant, as there are still challenges, some of which are beyond the researcher's control, involving social, or legislative issues that allow further investigation of the development and application of bioinoculants.

One challenge yet to be improved is the consistency of results, mainly in open field conditions or even in greenhouses where there is some control of climatic variables. There is a consensus to have several field applications for at least two or three seasons, providing better certainty about the results, not just a snapshot of a specific moment. This does not mean that such results are not important, but it is highly recommended to have as many repetitions as possible. Unfortunately, the pressures to publish and deliver quick results in many educational institutions force researchers not to wait for long-term field studies to be published in 5 or 10 years.

Building trust with producers is another challenge for all of us conducting research with microbe-based inoculants, including highly studied ones like *Trichoderma* and *Bacillus*. Other less studied microbial groups should also receive more attention since having more options from phylogenetically distinct groups broadens the horizon, providing more options in different edaphic and climatic regions. Particularly in saline soils, there is a consensus to use halophilic or at least halotolerant strains to predict greater survival of the inoculant. However, the remediation of these soils should also be a priority, which unfortunately is not carried out. Remediating saline soils (or with other problems) could expand the use and application of non-halotolerant groups that offer good options to improve crop production.

## 9 Conclusion

This work reviews pioneering and recent studies on two of the most reliable groups for interacting and improving plant growth and health, *Trichoderma* and *Bacillus*. However, there are still too many challenges to say that agrochemicals like fertilizers or microcides should be completely replaced. In some cases, from our point of view, a reduction in their use is already possible, and transitioning to more sustainable methods like bioinoculants is a reality. While some countries have promoted the use of bioinoculants, other regions are still in the process of accepting these new biotechnologies. Therefore, efforts must continue to build greater trust among agricultural producers with excellent products. Here, *Trichoderma* and *Bacillus* can play a fundamental role in achieving this goal globally.

## Author contributions

GS: Conceptualization, Supervision, Visualization, Writing – original draft, Writing – review & editing. MO-M: Writing – original draft, Writing – review & editing. MA: Writing – original draft, Writing – review & editing. DM: Writing – original draft, Writing – review & editing. EV-C: Writing – original draft, Writing – review & editing. LM-R: Formal analysis, Methodology, Writing – original draft, Writing – review & editing.
